# Associations of Multidimensional Image-Derived Facial Skin Scores with Adiposity Measures in a Large Screening Cohort in Japan: A Cross-Sectional Study with Internal Temporal Evaluation

**DOI:** 10.3390/jcm15218413

**Published:** 2026-10-29

**Authors:** Kimi Iinuma, Kazuyasu Fujii, Chisa Nakashima, Shunsuke Morita, Toshio Morikawa, Hidekazu Yamada, Ryuichi Morishita, Atsushi Otsuka

**Affiliations:** 1 Department of Dermatology, Kindai University Faculty of Medicine, 1-14-1 Mihara-dai, Minami-ku, Sakai 590-0197, Osaka, Japan; 2 Anti-Aging Center, Kindai University, 3-4-1 Kowakae, Higashiosaka 577-8502, Osaka, Japan; 3 Pharmaceutical Research and Technology Institute, Kindai University, 3-4-1 Kowakae, Higashiosaka 577-8502, Osaka, Japan; 4 Department of Clinical Gene Therapy, School of Medicine, Osaka University, 2-2 Yamada-oka, Suita 565-0871, Osaka, Japan

**Keywords:** adiposity, artificial intelligence, bioelectrical impedance, body composition, digital phenotyping, image-derived facial redness

## Abstract

**Background/Objectives**: Proprietary, age-referenced, image-derived facial skin scores may contain adiposity-related information, but their validity is uncertain. We tested whether 14 scores added predictive information for body mass index (BMI), bioelectrical impedance analysis (BIA)-derived body fat percentage, and a proprietary BIA-derived visceral fat level score beyond age category and sex, after redness, and, exploratorily, after BMI. **Methods**: This study included 387,859 deduplicated account IDs from a Japanese screening program, a 95.5% nested subset of the previous report’s unique-account cohort. Gradient-boosting models were developed using April–July data and evaluated without retuning on August–October data. Performance was assessed with R^2^, mean absolute error (MAE), and paired bootstrap confidence intervals. **Results**: In the internal temporal evaluation cohort (159,726 account IDs), the facial skin-score panel provided incremental predictive information beyond age category and sex and beyond redness. After BMI inclusion, however, the additional explained variance was negligible (ΔR^2^ = 0.00165 for body fat percentage and 0.00017 for the visceral fat level score). **Conclusions**: The panel provided modest, order-dependent information in internal temporal evaluation but negligible additional information after BMI inclusion. These unvalidated proprietary scores are hypothesis-generating and do not support diagnostic or body-compartment-specific use.

## 1. Introduction

Obesity affects cutaneous immune signaling, barrier function, microvascular physiology, and tissue mechanics [1,2,3,4,5]. Quantitative facial phenotypes may therefore contain adiposity-related information. Medical images can encode systemic features beyond their primary purpose: retinal photographs predict cardiovascular risk factors, facial photographs have been used to estimate body mass index (BMI), and three-dimensional facial imaging captures heterogeneity in ageing [6,7,8,9]. Commercial skin-analysis outputs, however, are age-referenced and are not equivalent to dermatologist-assessed signs; standardized acquisition and transparent evaluation are essential [10,11].

Our previous report used the same source dataset to examine univariable associations between BMI and each of the 14 image-derived facial skin scores, with detailed continuous, threshold-based, and obesity-category analyses centered on redness [12]. The present study addresses a distinct multivariable question: whether the scores jointly add information for BMI and bioelectrical impedance analysis (BIA)-derived outcomes beyond age category, recorded sex, redness, and, exploratorily, BMI. The contribution is therefore analytic rather than population-based, and the present cohort should not be interpreted as an independent replication.

The primary aim was to determine whether the 14 proprietary image-derived facial skin scores jointly provided incremental predictive information for BMI, BIA-derived body fat percentage, and the proprietary BIA-derived visceral fat level score beyond age category and recorded sex. Secondary aims were to quantify order-dependent incremental information after redness was entered first and, exploratorily, information remaining after BMI inclusion. Models were developed using April–July data and evaluated without retuning using August–October data from the same program as an internal temporal evaluation.

## 2. Materials and Methods

### 2.1. Study Design and Population

This cross-sectional secondary analysis used fully anonymized data from the Karada Measurement program conducted at the Osaka Healthcare Pavilion during Expo 2025 Osaka, Kansai, Japan, collected from April through October 2025. The source extract contained 484,462 assessment records. In the analytic dataset used for the present study, demographic age was represented as eight prespecified age categories (10s, 20s, 30s, 40s, 50s, 60s, 70s, and 80 years or older), rather than as exact individual-level age. The program included participants aged 18 years or older; consequently, the 10s category comprised participants aged 18–19 years. Age was therefore included in all models and adjusted analyses as a categorical covariate. Continuous skin-age, dental-age, and vascular-age indices, where available, were distinct device-derived age-like measures and were not used as demographic age. Primary eligibility required a nonmissing account identifier (ID), assessment month 4–10, recorded sex code 0 or 1, a valid age category, all 14 skin scores to be nonmissing and within the nominal 1–100 range, and BMI, body fat percentage, and the visceral fat level score to be nonmissing and greater than 0. Because only measurement month, rather than full date and time, was available, eligible records were ordered by account ID, measurement month, and source-row number. The first eligible record under this deterministic ordering was retained for each account ID. Deduplication was performed at the account-ID level rather than using verified biological identity. Although repeated records within the same account were removed, one individual could potentially have held multiple accounts; therefore, residual duplication and non-independence at the person level cannot be completely excluded. The earlier publication analyzed 410,384 assessment records. An account-ID reanalysis of that earlier analytic file identified 406,338 unique account IDs. All 387,859 account IDs in the present study were represented among those unique IDs, corresponding to 95.5% at the account-ID level; the present cohort was therefore not an independent population.

Account IDs first eligible in April–July formed the development sample, and those first eligible in August–October formed the internal temporal evaluation sample. Before deduplication, 547 eligible account IDs had records in both periods; assignment according to the first eligible record ensured that no account ID overlapped between samples. Development IDs were split 80:20 into stratified training and validation sets. The Ethics Committee of Kindai University Faculty of Medicine approved this secondary analysis on 2 February 2026 (approval no. R07-210). All participants had provided informed consent at the time of data collection. Reporting was guided by relevant TRIPOD+AI items [13].

### 2.2. Measurements

Facial images were assessed using the commercial AI Skin Diagnostic system (Perfect Corp., New York, NY, USA), which generated 14 proprietary, age-referenced scores: pigmentation (spots), wrinkles, texture, oiliness, redness, eye bags, pores, moisture, upper-eyelid sagging, lower-eyelid sagging, firmness, radiance, dark circles, and acne. Program documentation described each score as an image-based evaluation relative to the vendor-defined mean for the corresponding age group; a score near 80 denoted that age-group average, and higher scores indicated more favorable findings. The available documentation did not specify the age-band boundaries, whether reference values were additionally stratified by sex or other characteristics, or the normalization equations. Thus, a lower native redness score indicated greater image-derived facial redness. The outputs were treated as nominal 1–100 scores and were not equivalent to dermatologist-assessed diagnoses. The manufacturer documentation available to the investigators did not report the model architecture, training data, test–retest reliability, inter-image reproducibility, agreement with dermatologist assessment, reference-age intervals, normalization equations, or other technical-validation results. No additional validation documentation was available for this analysis. Accordingly, the outputs were treated as proprietary image-derived scores rather than validated clinical phenotypes, and their precise construct meaning and independent reproducibility could not be established. Contemporaneous records of the imaging hardware, software version, scoring configuration, acquisition conditions, formal quality-control procedures, participant preparation, and fields passed to the skin-scoring system were unavailable. Consequently, whether BMI or BIA outputs were excluded from the skin-scoring workflow could not be verified. The investigators were not aware of deliberate changes between the development and evaluation periods; however, unchanged hardware, software, scoring parameters, and acquisition conditions could not be verified. Body composition was assessed during the same visit using a Tanita DC-13C professional bioelectrical-impedance analyzer adapted for the program. The documented inputs and measurements used for body-composition estimation were age, sex, height, measured weight, and impedance. BMI was calculated from measured weight and recorded height. Body fat percentage was an indirect BIA-derived estimate of fat mass as a percentage of body weight and should not be considered interchangeable with criterion body-composition measurements [14]. The visceral fat level score was a proprietary BIA-derived index and did not represent a direct measurement of visceral adipose tissue area or volume. Agreement between BIA-derived intra-abdominal fat estimates and computed tomography or magnetic resonance imaging is limited at the individual level [15], although such indices have been evaluated for metabolic-risk screening [16]. The vendor’s age referencing and the study’s categorical age adjustment were therefore distinct: the former was embedded in proprietary scores using undisclosed age bands and normalization, whereas the latter used the eight demographic age categories available in the analytic dataset. Because these age-band definitions could not be aligned, categorical adjustment cannot be assumed to remove all age-related structure embedded in the skin scores.

### 2.3. Analytic Models and Internal Temporal Evaluation

Analyses were performed using Python version 3.14.4 (Python Software Foundation, Wilmington, DE, USA) with LightGBM version 4.7.0 (Microsoft Corporation, Redmond, WA, USA), scikit-learn version 1.9.0, pandas version 3.0.3, NumPy version 2.5.1, SciPy version 1.18.0, statsmodels version 0.14.6, and matplotlib version 3.11.1.

For each body-composition outcome, Light Gradient Boosting Machine (LightGBM) models included age category and recorded sex (M0), M0 plus redness (MR), or age category, recorded sex, and all 14 skin scores (M2) [17]. The primary models therefore used age category and recorded sex, with or without the precomputed skin scores; the adiposity outcomes were not included as predictors in these models. BMI was included only in the explicitly designated exploratory BMI-informed analyses of the BIA-derived outcomes. Base settings used a learning rate of 0.05, 63 leaves, a minimum of 100 observations per leaf, and deterministic column-wise fitting. Up to 1000 trees were considered, and the number of trees was selected separately for each outcome and model using only the development validation set. The selected numbers of trees for all final models, including the exploratory BMI-informed models, are reported in Supplementary Table S1. Final models were refitted using all development IDs and applied without retuning to the internal temporal evaluation sample. This out-of-period prediction framework was used to quantify incremental information rather than to develop a deployable clinical prediction tool.

Performance was quantified using R^2^ and mean absolute error (MAE). Percentile 95% confidence intervals (CIs) were generated from 1000 bootstrap samples of unique evaluation account IDs. Within each replicate, the same sampled IDs were applied to predictions from all compared models, permitting paired CIs for differences in R^2^. These CIs were conditional on the fitted models and did not include model-development uncertainty. Relative residual improvement was calculated as ΔR^2^/(1 − R^2^ of the baseline model) to support comparison across outcomes with different baseline R^2^ values. Detailed calibration procedures, definitions, units, and plots are provided in the Supplementary Methods, Supplementary Table S4, and Supplementary Figure S2. Frozen-model predictions were used without model refitting or retuning.

### 2.4. Feature and Incremental Analyses

Mean absolute tree-based Shapley additive explanations (TreeSHAP) contributions were computed from each final M2 model in 10,000 evaluation account IDs to rank predictive features [18]. TreeSHAP values were computed using LightGBM’s built-in pred_contrib implementation; the standalone SHAP package was not used. Spearman correlations among the 14 skin scores were calculated in the internal temporal evaluation cohort. Directional associations were examined in 42 separate standardized linear regression models, with one body-composition outcome as the dependent variable and one skin score as the predictor. Models were adjusted for 16 age-category-by-recorded-sex strata and measurement-month fixed effects, and heteroskedasticity-consistent type 1 (HC1) robust 95% CIs were calculated. *p* values were adjusted across all 42 tests using the Benjamini–Hochberg false discovery rate procedure. Because correlated predictors can share or redistribute model importance, TreeSHAP rankings and directional coefficients were interpreted descriptively rather than as independent or causal effects. Sequential incremental analyses compared MR with M0 and M2 with MR. Because redness was entered before the remaining 13 scores, these increments were order-dependent and were not interpreted as unique variance attributable to redness or to the remaining scores. In exploratory BMI-informed analyses, all 14 skin scores were added to baseline LightGBM models containing age category, recorded sex, and BMI for body fat percentage and the visceral fat level score. The baseline and expanded models were developed using April–July data and evaluated without retuning using the same August–October cohort. Differences in R^2^ were evaluated using 1000 paired bootstrap samples. Because the evaluation dataset contained one record per deduplicated account ID, resampling evaluation rows was equivalent to account-ID-level resampling. Sensitivity analyses examined account IDs with a single eligible record, rank-based inverse normal transformation (RINT) of redness, and recorded-sex strata.

To assess selection associated with complete-case eligibility, we compared the 391,482 eligible source records with the 92,980 ineligible source records before account-ID deduplication. Comparisons were descriptive and used age category, recorded sex, and available body-composition measures. Categorical variables were summarized as counts and percentages and continuous variables as mean (SD). Standardized mean differences (SMDs) were calculated for the reported binary age and recorded-sex contrasts and for continuous comparisons. Body-composition comparisons in the ineligible group were restricted to nonmissing values, and their missingness was reported.

## 3. Results

### 3.1. Analytic Sample

**Figure 1:**
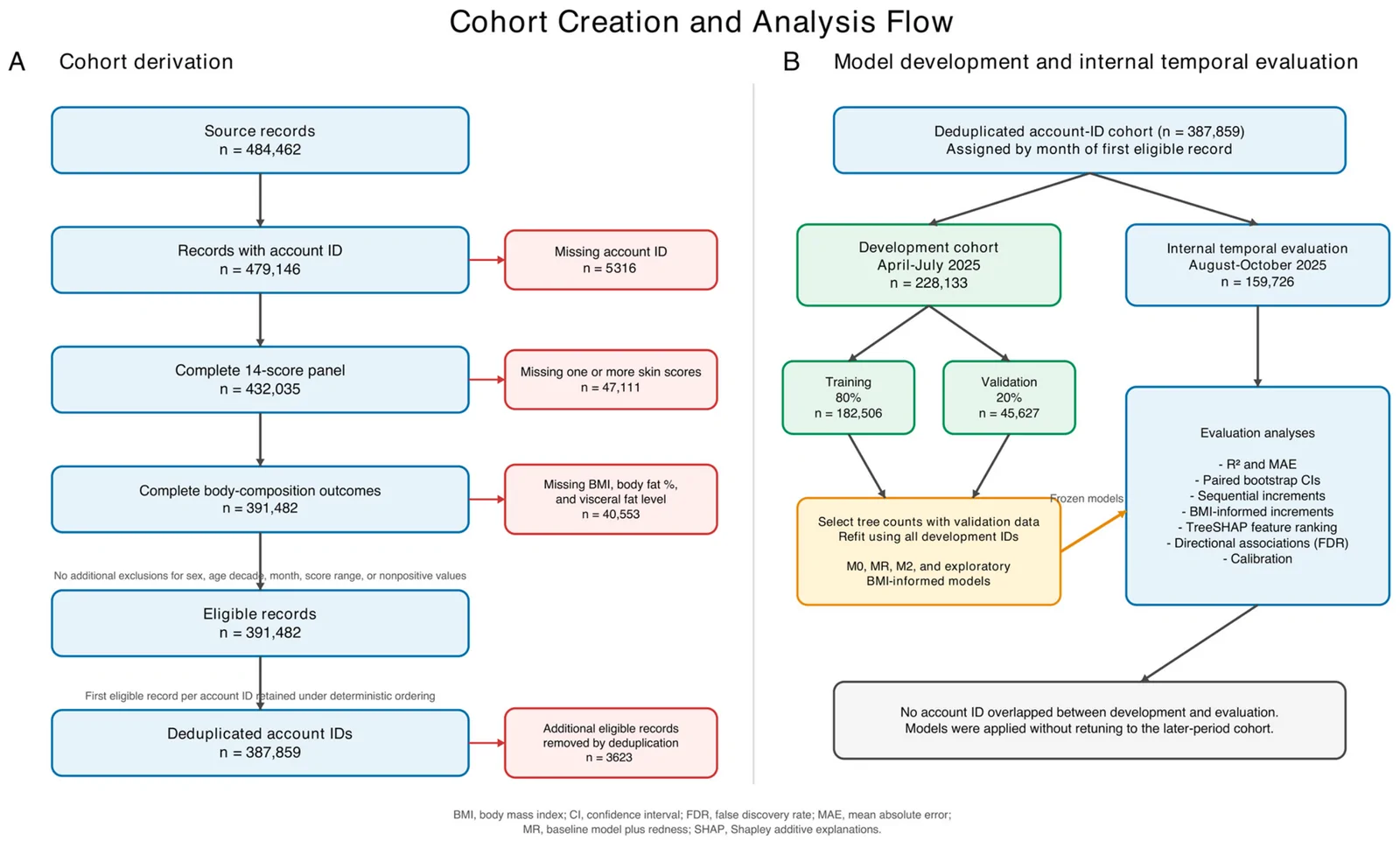
Cohort creation and analysis flow. (**A**) Cohort derivation and account-ID deduplication. (**B**) Temporal cohort allocation, model development, and internal temporal evaluation. Gray arrows indicate record or analysis flow, red arrows indicate exclusions, and the orange arrow indicates the application of frozen models to the evaluation analyses.

Of 484,462 source records, 391,482 met complete-case eligibility. The principal exclusions were a missing account ID (*n* = 5316), one or more missing skin scores (*n* = 47,111), and failure to meet the complete body-composition criterion for BMI, body fat percentage, and the visceral fat level score at the corresponding sequential exclusion step (*n* = 40,553). After deterministic selection of one eligible record per account ID, 387,859 account IDs remained (Figure 1A). Of these, 228,133 were assigned to model development and 159,726 to internal temporal evaluation (Figure 1B). No account ID overlapped between the development and evaluation samples. The evaluation sample was 61.9% female, with a mean (SD) BMI of 22.3 (3.8), body fat percentage of 25.0% (8.0%), visceral fat level score of 6.4 (4.3), and redness score of 87.1 (9.2) (Table 1). Redness and oiliness showed substantial upper-end heaping, with 19.3% and 17.9% of observations, respectively, at their observed maxima of 99. Several other scores had restricted observed ranges or skewed distributions (Table 2).

In the included-versus-excluded comparison, eligible records were less often female than ineligible records (60.6% vs. 67.2%; SMD, −0.136). Records from participants aged 70 years or older comprised 2.2% of eligible records and 6.3% of ineligible records (SMD, −0.205), whereas records from participants in their 30s through 50s were more frequent among eligible records. Among the 43,063 ineligible records with nonmissing body-composition values, mean (SD) BMI was 21.90 (3.90) kg/m^2^ compared with 22.34 (3.83) kg/m^2^ among eligible records (SMD, 0.115); corresponding values were 25.60% (8.20%) versus 25.12% (8.01%) for body fat percentage (SMD, −0.059) and 5.77 (4.12) versus 6.55 (4.35) device-score units for the visceral fat level score (SMD, 0.185). Body-composition values were missing in 53.7% of ineligible records; therefore, these continuous-variable comparisons describe only the nonmissing subset and do not exclude selection related to missing or unmeasured characteristics.

### 3.2. Model Performance in Internal Temporal Evaluation

**Figure 2:**
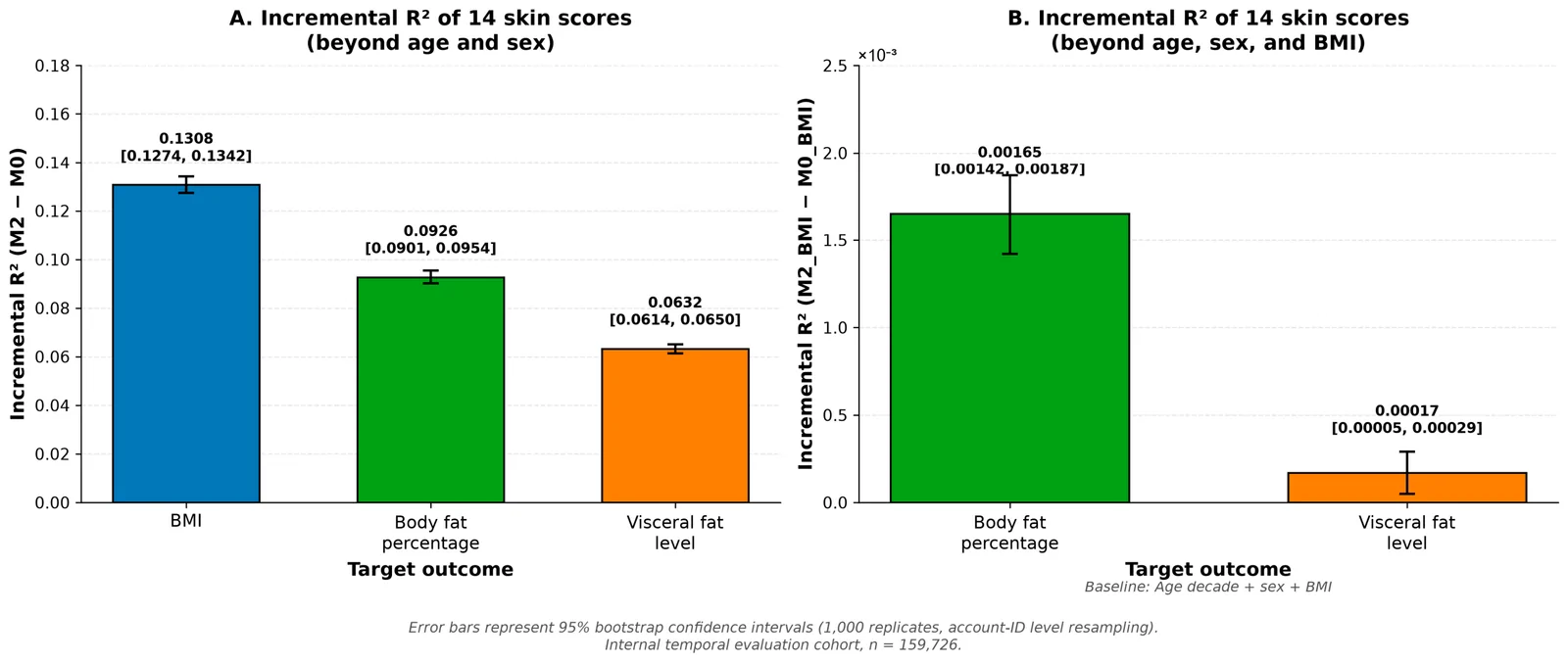
Incremental information from the 14-score facial skin panel before and after BMI inclusion. (**A**) Incremental R^2^ when all 14 skin scores were added to models containing age category and recorded sex (M2 vs. M0). (**B**) Incremental R^2^ when all 14 skin scores were added to exploratory BMI-informed baseline models for body fat percentage and visceral fat level score. Error bars indicate 95% confidence intervals obtained from 1000 paired account-ID bootstrap samples in the internal temporal evaluation cohort (*n* = 159,726).

Adding all 14 skin scores to age category and recorded sex increased evaluation-sample R^2^ from 0.125 to 0.256 for BMI (ΔR^2^, 0.131; 95% CI, 0.127–0.134), from 0.254 to 0.347 for body fat percentage (ΔR^2^, 0.093; 95% CI, 0.090–0.095), and from 0.549 to 0.612 for visceral fat level score (ΔR^2^, 0.063; 95% CI, 0.061–0.065) (Table 3). Corresponding MAE values decreased from 2.713 to 2.500 kg/m^2^, from 5.376 to 5.036 percentage points, and from 2.198 to 2.043 device-score units, respectively. Relative to residual variance under M0, the R^2^ improvements were 15.0%, 12.5%, and 14.0%, respectively (Table 3 and Figure 2A).

Results were similar when the evaluation sample was restricted to account IDs with a single eligible record (*n* = 159,125; M2 R^2^: BMI, 0.256; body fat percentage, 0.347; visceral fat level score, 0.612) (Supplementary Table S5, Panel A).

### 3.3. Order-Dependent Information After Redness and Information After BMI Inclusion

In the model sequence with redness entered first, adding redness to age category and recorded sex increased R^2^ by 0.064 for BMI, 0.046 for body fat percentage, and 0.028 for visceral fat level score. Subsequent addition of the remaining 13 scores further increased R^2^ by 0.067, 0.046, and 0.035, respectively (Table 3). Because the skin scores were correlated, these sequential increments are order-dependent and should not be interpreted as unique variance attributable to redness or to the remaining scores.

In exploratory BMI-informed analyses, baseline R^2^ values were 0.86927 for body fat percentage and 0.96156 for the visceral fat level score. Adding all 14 skin scores increased R^2^ to 0.87092 for body fat percentage (ΔR^2^ = 0.00165; 95% CI, 0.00142–0.00187) and 0.96173 for the visceral fat level score (ΔR^2^ = 0.00017; 95% CI, 0.00005–0.00029) (Figure 2B). MAE changed from 2.194 to 2.183 percentage points for body fat percentage and from 0.526 to 0.532 device-score units. Thus, the statistically detectable increments in R^2^ did not correspond to consistent improvements across performance metrics and were practically negligible. The high performance of the BMI-informed models may partly reflect shared anthropometric inputs or proprietary estimation procedures and should not be interpreted as accurate prediction of criterion-measured body compartments (Supplementary Table S2).

### 3.4. Contributing Skin Scores and Directional Associations

Redness had the largest mean absolute TreeSHAP value among the facial skin scores for all three outcomes (Table 4). Other leading features included texture, moisture, oiliness, firmness, and upper-eyelid sagging. Because correlated predictors can share or redistribute attribution, these rankings describe the fitted models and do not establish unique, independent, or biological contributions of any individual score.

In adjusted directional analyses, higher native redness scores were associated with lower BMI (standardized β = −0.258), body fat percentage (β = −0.216), and visceral fat level score (β = −0.172) (Supplementary Table S3). Because lower redness scores indicate greater image-derived facial redness, these results correspond to higher adiposity measures among participants with greater facial redness. Complete coefficients and multiplicity-adjusted *p* values for the 42 directional analyses are reported in Supplementary Table S3. Given the large sample, statistical significance was not interpreted as evidence of scientific or practical importance. Correlations among the skin scores were variable, with the strongest correlation observed between texture and moisture (Spearman ρ = 0.839; Supplementary Figure S1).

Calibration slopes were close to 1.0 (1.008–1.016), and decile-level MCE values ranged from 0.058 to 0.088; these summaries did not establish individual-level accuracy. Redness RINT sensitivity analyses were directionally concordant, and incremental R^2^ estimates were positive in both recorded-sex strata; these secondary results are detailed in Supplementary Tables S4 and S5 and Supplementary Figure S2.

## 4. Discussion

After BMI inclusion, adding all 14 skin scores increased R^2^ by only 0.00165 for body fat percentage and 0.00017 for the visceral fat level score; MAE improved minimally for body fat percentage and worsened slightly for the visceral fat level score. These changes were practically negligible and do not establish clinically meaningful prediction or support clinical use. The larger, order-dependent increments observed before BMI inclusion should therefore be interpreted as exploratory predictive associations rather than evidence of diagnostic value. The findings do not support using facial skin scores to replace routine anthropometric measurements or reference-standard body-composition assessment. At most, these scores may be considered hypothesis-generating digital phenotypes [19] for studying possible cutaneous–systemic relationships, not diagnostic tests or body-compartment-specific health-risk indicators.

Although BIA estimation was documented to use age, sex, height, measured weight, and impedance, the fields supplied to the proprietary skin-scoring system were unavailable. We therefore could not determine whether anthropometric variables, BMI, or BIA-derived information contributed directly or indirectly to skin-score generation. Such input overlap could produce information coupling or leakage and inflate apparent predictive performance. Accordingly, the results cannot be interpreted as demonstrating information derived exclusively from facial images, independent of those inputs. The image-derived measurements should therefore be regarded as unvalidated proprietary outputs whose independence from anthropometric and BIA-derived information remains unverified.

The earlier publication analyzed 410,384 assessment records. An account-ID reanalysis of that earlier analytic file identified 406,338 unique account IDs, and all 387,859 account IDs in the present study were represented among them (95.5%). The previous report examined univariable associations between BMI and the 14 scores, with detailed analyses centered on redness. The present study instead evaluated the 14 scores jointly, added BIA-derived body fat percentage and the proprietary BIA-derived visceral fat level score as outcomes, quantified order-dependent incremental information after redness and information remaining after BMI inclusion, and evaluated models in a later nonoverlapping period without retuning. The genuinely new findings are that the combined panel provided modest incremental prediction beyond age and sex, whereas its additional predictive information for the BIA-derived outcomes was negligible after BMI was included. The reports therefore address complementary analytic questions in nested cohorts rather than independent populations. Accordingly, the scientific contribution beyond the previous publication is the joint and sequential predictive evaluation across three outcomes; it is not independent replication or validation of the image-derived measurements.

These analyses address association and prediction, not biological mechanism. Correlations and TreeSHAP rankings may reflect facial morphology, adiposity-related appearance, lighting or other acquisition conditions, cosmetics, age-related changes, or unmeasured behavioral and clinical factors; they do not identify independent determinants or causal pathways. Adiposity is associated with inflammatory signaling, altered skin-barrier and hydration measures, microvascular dysfunction, and mechanical changes in skin [1,2,3,4,5,20], and facial appearance may also encode age- and lifestyle-related variation [9]. However, the internal temporal evaluation cohort was relatively lean (mean BMI, 22.3 kg/m^2^), so mechanisms described in obesity cannot be assumed to explain the associations across this BMI distribution. The recurrence of redness, texture, moisture, and oiliness across outcomes is compatible with several biological and image-related explanations but is not mechanistically specific. Rosacea has been associated with cardiometabolic comorbidities in a systematic review and meta-analysis [21]; however, the proprietary redness score was not validated against clinical erythema in this dataset and was not established as an erythema measure. It is neither a diagnosis of rosacea nor evidence of inflammation or a specific microvascular mechanism; dermatologic diagnoses were unavailable. Because exact individual-level age and the vendor’s normalization procedure were unavailable, categorical age adjustment could neither reproduce nor remove the vendor’s age referencing; residual age-related information within categories and within the proprietary scores cannot be excluded, and sensitivity analysis using flexibly modeled continuous age could not be performed.

Strengths include the large sample, ID-level deduplication, nonoverlapping temporal development and evaluation periods, paired bootstrap comparisons, feature-importance and directional analyses, and explicit assessment of order-dependent increments after redness and incremental information after BMI inclusion. The relative-residual-improvement analysis reduced the risk of misleading comparisons arising from different baseline R^2^ values across outcomes. Sensitivity analyses were directionally consistent across recorded-sex strata.

Several limitations remain. The cross-sectional, complete-case design in a single relatively lean Japanese screening cohort limits causal inference and generalizability. Missingness may have been nonrandom, and participant-level ethnicity and important behavioral and clinical covariates were unavailable. Although included and excluded records were compared, substantial missingness among ineligible records prevented exclusion of selection related to missing or unmeasured factors. Although one eligible record was retained per account ID and no account ID was shared between the development and evaluation cohorts, account IDs were not verified person-level identifiers. Multiple accounts belonging to the same individual, and the resulting residual dependence, cannot be completely excluded. The proprietary, age-referenced skin scores were not validated against dermatologist-assessed findings, and incomplete documentation of their normalization, system inputs, software implementation, acquisition conditions, and quality control limits independent reproduction and assessment of generalizability across settings, devices, software versions, and populations. Reproducibility of the downstream statistical analyses therefore does not establish measurement validity or clinical utility. Body fat percentage and the visceral fat level score were indirect device estimates rather than criterion measurements. Score heaping and restricted ranges could limit discrimination among participants with tied or compressed values and make tree splits and SHAP rankings sensitive to the observed score distributions; the feature rankings should therefore be interpreted as dataset- and model-specific. Finally, evaluation used adjacent periods within the same program rather than an external cohort, and the bootstrap confidence intervals excluded model-development uncertainty.

## 5. Conclusions

Combined proprietary, age-referenced, image-derived facial skin scores contained order-dependent incremental information after redness was entered first and retained this pattern in internal temporal evaluation. However, they provided negligible additional predictive information for BIA-derived body fat percentage and the proprietary BIA-derived visceral fat level score after BMI was included. Accordingly, the findings do not support using facial skin scores to replace routine anthropometric measurements or reference-standard body-composition assessment. The scores remain hypothesis-generating, and the results do not establish validated clinical skin phenotypes, biological mechanisms, external generalizability, diagnostic utility, or body-compartment-specific prediction.

## Figures and Tables

**Table 1:** Characteristics of the deduplicated analytic sample.

Characteristic	Development April–July	Evaluation August–October	Overall
Account IDs, *n*	228,133	159,726	387,859
Recorded female sex, *n* (%)	135,863 (59.6)	98,851 (61.9)	234,714 (60.5)
Age 18–39 years, *n* (%)	85,139 (37.3)	67,699 (42.4)	152,838 (39.4)
Age 40–59 years, *n* (%)	104,940 (46.0)	71,727 (44.9)	176,667 (45.5)
Age ≥60 years, *n* (%)	38,054 (16.7)	20,300 (12.7)	58,354 (15.0)
BMI, mean (SD)	22.4 (3.8)	22.3 (3.8)	22.3 (3.8)
Body fat percentage, mean (SD)	25.2 (8.0)	25.0 (8.0)	25.1 (8.0)
Visceral fat level score, mean (SD)	6.7 (4.4)	6.4 (4.3)	6.6 (4.4)
Redness score, mean (SD)	87.0 (9.2)	87.1 (9.2)	87.1 (9.2)

Values are *n* (%) unless otherwise indicated. For descriptive presentation, the eight source age categories were pooled into 18–39, 40–59, and ≥60 years; participants in the source 10s category were aged 18–19 years because the program included participants aged 18 years or older. Models and adjusted analyses used the original eight source age categories. Exact continuous age was unavailable in the present analytic extract. The visceral fat level score is a proprietary BIA-derived index, not a direct measurement of visceral adipose tissue. BMI, body mass index; SD, standard deviation.

**Table 2:** Distribution of 14 image-derived facial skin scores in the internal temporal evaluation sample.

Skin Score	Observed Range	Mean (SD)	Median [IQR]	At Observed Maximum, *n* (%)	Skewness
Pigmentation	63–97	86.1 (7.8)	88.0 [79.0–93.0]	5895 (3.7)	−0.34
Wrinkle	54–98	90.0 (7.5)	93.0 [86.0–96.0]	1982 (1.2)	−1.15
Texture	56–99	82.5 (9.2)	81.0 [77.0–90.0]	88 (0.1)	−0.18
Oiliness	70–99	90.1 (8.0)	92.0 [84.0–98.0]	28,655 (17.9)	−0.59
Redness	53–99	87.1 (9.2)	86.0 [80.0–96.0]	30,888 (19.3)	−0.23
Eye bag	57–99	82.9 (8.1)	83.0 [79.0–88.0]	1433 (0.9)	−0.26
Pore	57–97	78.3 (8.6)	78.0 [72.0–84.0]	2504 (1.6)	+0.37
Moisture	62–85	74.6 (3.8)	75.0 [72.0–77.0]	229 (0.1)	−0.34
Upper eyelid sagging	52–94	81.4 (6.1)	83.0 [80.0–84.0]	822 (0.5)	−1.41
Lower eyelid sagging	56–84	73.1 (5.2)	73.0 [69.0–77.0]	207 (0.1)	−0.28
Firmness	54–94	80.3 (7.8)	82.0 [77.0–85.0]	533 (0.3)	−0.95
Radiance	54–95	81.6 (6.9)	82.0 [77.0–86.0]	1643 (1.0)	−0.40
Dark circle	70–91	80.6 (3.8)	80.0 [78.0–83.0]	542 (0.3)	+0.39
Acne	74–99	83.7 (5.9)	82.0 [79.0–87.0]	6761 (4.2)	+0.97

Evaluation sample *n* = 159,726 for every score. The observed maximum was score-specific and ranged from 84 to 99; therefore, the percentage at the observed maximum describes upper-end heaping and is not the percentage at the nominal scale ceiling of 100. IQR, interquartile range; SD, standard deviation.

**Table 3:** Predictive performance of the LightGBM models in the internal temporal evaluation cohort.

(**A**) **R^2^ and Incremental R^2^**				
**Outcome**	**M0 R^2^ [95% CI]**	**MR R^2^ [95% CI]**	**M2 R^2^ [95% CI]**	**M2 − M0 ΔR^2^ [95% CI]**
BMI	0.125 [0.122, 0.128]	0.189 [0.186, 0.193]	0.256 [0.252, 0.260]	+0.131 [+0.127, +0.134]
Body fat percentage	0.254 [0.251, 0.257]	0.300 [0.296, 0.304]	0.347 [0.343, 0.351]	+0.093 [+0.090, +0.095]
Visceral fat level score	0.549 [0.545, 0.553]	0.577 [0.573, 0.581]	0.612 [0.609, 0.616]	+0.063 [+0.061, +0.065]
(**B**) **Mean Absolute Error**				
**Outcome**	**M0 MAE [95% CI]**	**MR MAE [95% CI]**	**M2 MAE [95% CI]**	**M2 − M0 Change**
BMI	2.713 [2.701, 2.724]	2.614 [2.604, 2.625]	2.500 [2.490, 2.511]	−0.213
Body fat percentage	5.376 [5.354, 5.397]	5.216 [5.196, 5.236]	5.036 [5.017, 5.056]	−0.340
Visceral fat level score	2.198 [2.188, 2.207]	2.130 [2.121, 2.138]	2.043 [2.035, 2.051]	−0.155

Evaluation sample *n* = 159,726. M0 included age category and recorded sex; MR included M0 plus redness; and M2 included M0 plus all 14 facial skin scores. All models used LightGBM, were developed using April–July data, and were applied without retuning to the August–October evaluation sample. Confidence intervals are conditional on the fitted models. Body fat percentage is a BIA-derived estimate, and the visceral fat level score is a proprietary BIA-derived index; neither is a criterion measurement of a specific body compartment. BMI, body mass index; CI, confidence interval; MAE, mean absolute error. Sequential ΔR^2^ values were calculated from unrounded model estimates; therefore, displayed component values may not sum exactly to the displayed total because of rounding.

**Table 4:** Leading facial skin features in the M2 models during internal temporal evaluation.

Outcome	Skin Rank	Skin Feature	Mean |SHAP|	Share, %
BMI	1	Redness	0.7622	18.2
	2	Texture	0.4832	11.5
	3	Moisture	0.3993	9.5
	4	Oiliness	0.3635	8.7
	5	Upper-eyelid sagging	0.2581	6.2
Body fat percentage	1	Redness	1.2698	11.9
	2	Oiliness	0.7234	6.8
	3	Texture	0.6759	6.3
	4	Moisture	0.6736	6.3
	5	Firmness	0.4749	4.4
Visceral fat level score	1	Redness	0.5539	9.4
	2	Moisture	0.3130	5.3
	3	Oiliness	0.3064	5.2
	4	Texture	0.2984	5.1
	5	Upper-eyelid sagging	0.1791	3.0

Mean absolute SHAP values were calculated from the final M2 model in a random sample of 10,000 account IDs from the internal temporal evaluation cohort. M2 included age category, recorded sex, and all 14 facial skin scores. SHAP values are expressed in each outcome’s native scale and should not be compared across outcomes. Share represents the proportion of the total mean absolute SHAP magnitude across all M2 predictors, including age category and recorded sex. Because correlated features may share or redistribute model importance, the rankings are descriptive and should not be interpreted as independent or causal effects. BMI, body mass index; SHAP, Shapley additive explanations.

## Data Availability

The data that support the findings of this study are available from the corresponding author upon reasonable request.

## Supplementary Materials

The following supporting information can be downloaded at: https://www.mdpi.com/article/10.3390/jcm15218413/s1, Figure S1: Spearman correlation matrix of the 14 facial skin scores in the internal temporal evaluation cohort; Figure S2: Calibration of the final M2 models in the internal temporal evaluation cohort; Table S1: Selected numbers of trees for the final LightGBM models; Table S2: Performance of exploratory BMI-informed models in the internal temporal evaluation cohort; Table S3: Adjusted directional associations between individual facial skin scores and body-composition outcomes; Table S4: Calibration of the final M2 models in the internal temporal evaluation cohort; Table S5: Sensitivity analyses.

## Abbreviations and Acronyms

AI, artificial intelligence; BIA, bioelectrical impedance analysis; BMI, body mass index; CI, confidence interval; HC1, heteroscedasticity-consistent type 1; ID, identifier; IQR, interquartile range; MAE, mean absolute error; RINT, rank-based inverse normal transformation; SD, standard deviation; SHAP, Shapley additive explanations.
