# Supplementary material for: Associations of Multidimensional Image-Derived Facial Skin Scores with Adiposity Measures in a Large Screening Cohort in Japan: A Cross-Sectional Study with Internal Temporal Evaluation
Source: J Clin Med. 2026 Oct 29;15(21):8413. doi: 10.3390/jcm15218413 (PMC13763775; doi:10.3390/jcm15218413)
Supplement: Supplementary file 1 [file jcm-15-08413-s001.zip › jcm-4599431-supplementary.pdf]

Supplementary Material

Associations of Multidimensional Image-Derived Facial Skin Scores With Adiposity Measures in a Large Screening Cohort in Japan: A Cross-Sectional Study With Internal Temporal Evaluation

Kimi Iinuma, MD; Kazuyasu Fujii, MD, PhD; Chisa Nakashima, MD, PhD; Shunsuke Morita, MD; Toshio Morikawa, PhD; Hidekazu Yamada, MD, PhD; Ryuichi Morishita, MD, PhD; Atsushi Otsuka, MD, PhD

Supplementary Methods

Study design and source population

This cross-sectional study used fully anonymized data from the Karada Measurement program conducted at the Osaka Healthcare Pavilion during Expo 2025 Osaka, Kansai, Japan. The extract contained 484,462 measurement records from April through October 2025. Deduplication was performed at the account-ID level rather than using verified biological identity. Although repeated records within the same account were removed, one individual could potentially have held multiple accounts; therefore, residual duplication and non-independence at the person level cannot be completely excluded.

Facial skin and body-composition measurements

Facial images were assessed using the AI Skin Diagnostic system (Perfect Corp.). Contemporaneous records of the imaging hardware, software version, scoring configuration, acquisition conditions, formal quality-control procedures, participant preparation, and fields passed to the skin-scoring system were unavailable. Therefore, standardized lighting, positioning, and other acquisition conditions could not be independently verified. The 14 age-referenced component scores analyzed in this study were pigmentation (spots), wrinkles, texture, oiliness, redness, eye bags, pores, moisture, upper-eyelid sagging, lower-eyelid sagging, firmness, radiance, dark circles, and acne. Program documentation described each score as an image-based evaluation relative to the vendor-defined mean for the corresponding age group; a score near 80 denoted that age-group average, and higher scores indicated more favorable findings. The available documentation did not specify age-band boundaries, whether reference values were additionally stratified by sex or other characteristics, or the normalization equations. Accordingly, 80 was not treated as a universal clinical norm or independently validated reference value. A lower redness score was therefore interpreted as greater image-derived facial redness. Consistent with the previous report, the 14 outputs were treated as nominal 1–100 scores. The vendor did not disclose the

model architecture, training data, reliability coefficients, reference-age intervals, or normalization equations. Independent reconstruction and external validation of the 14 scores were therefore not possible using the available documentation. No additional vendor validation documentation was available for the present analysis. For data-quality checking, eligibility required values within 1–100. All nonmissing values met this criterion, and observed values in the source extract ranged from 52 to 99. The previous BMI–redness report analyzed a reverse-coded redness score (101 − native score), whereas the present study retained the native score; therefore, positive associations in the previous report correspond to negative coefficients in the present analysis. [12]

These scores were not dermatologist-assessed diagnoses. Cosmetics and other external factors were not fully standardized, and original facial images were unavailable in the anonymized analysis extract. Standardization of lighting, color, positioning, image metadata, and algorithm evaluation is central to the reliability and generalizability of dermatologic imaging studies. [10], [11] Because the vendor's reference-age intervals and normalization equations were unavailable, we could not determine how its age-referencing procedure related to the eight demographic age categories used in our models; categorical age adjustment therefore does not establish age-independent information, and residual or differently encoded age-related information may remain in the scores. In addition, direct or indirect use of anthropometric variables, BMI, or BIA-derived information in skin-score generation could not be excluded; such upstream overlap could produce information coupling or leakage and inflate apparent predictive performance.

Body composition was measured during the same visit using a Tanita DC-13C hand-contact bioelectrical-impedance analyzer adapted for the pavilion program. The documented inputs and measurements used for body-composition estimation were age, sex, height, measured weight, and impedance. The present analysis used age categories for adjustment and model development. BMI was calculated from measured weight and recorded height. Body fat percentage was a BIA-derived estimate of fat mass as a percentage of body weight. Because BIA is an indirect, device- and protocol-dependent method, these estimates should not be considered interchangeable with criterion body-composition measurements. [14] The visceral fat level score was a proprietary BIA-derived index of estimated visceral fat accumulation rather than a direct measurement of abdominal visceral adipose tissue; agreement of BIA-derived intra-abdominal fat estimates with computed tomography or magnetic resonance imaging is limited at the individual level. [15] The proprietary equations

used to derive body fat percentage and the visceral fat level score were unavailable, and the extent to which these outcomes shared anthropometric inputs with BMI could not be quantified.

Eligibility, deduplication, and split

Primary eligibility required a nonmissing account ID; measurement month 4–10; recorded sex code 0 or 1; a valid age category; all 14 skin scores to be nonmissing and within the nominal 1–100 range; and BMI, body fat percentage, and visceral fat level score to be nonmissing and greater than 0. Criteria were applied sequentially. Of 484,462 source records, 5,316 were excluded because of a missing account ID. No additional records were excluded because of invalid recorded sex, age-category, or measurement-month values. A further 47,111 records were excluded because at least one of the 14 skin scores was missing; no additional record had a skin-score value outside 1–100. Among the remaining records, 40,553 were excluded because they failed the complete body-composition criterion: at least one of BMI, body fat percentage, or the visceral fat level score was missing. No additional record had a nonpositive value for any of these three outcomes. This process yielded 391,482 eligible records before account-ID deduplication. Because only measurement month, rather than full date and time, was available, eligible records were ordered by account ID, measurement month, and source-file row number. The first eligible record under this deterministic ordering was retained for each account ID. Source-file row number served as the tie-breaker for multiple eligible records occurring within the same month. Deduplication yielded 387,859 account IDs, of which 228,133 were assigned to the development sample and 159,726 to the internal temporal evaluation sample. Account IDs first eligible in April–July formed the development data; account IDs first eligible in August–October formed the internal temporal evaluation data. Development account IDs were split 80:20 into training and validation samples, stratified by month, age category, and recorded sex. No account ID overlapped across splits.

Relationship to the previous report and account-ID sensitivity analysis

The previous report used 410,384 records from 406,338 unique account IDs from the same 484,462-record source extract. That analysis required 10 < BMI < 60 and a nonmissing native redness score within the nominal 1–100 range but did not require complete data for all 14 skin scores. The present analysis did not impose the same BMI-range restriction; instead, it required nonmissing positive values for BMI, body fat percentage, and visceral fat level score, together with complete values within the nominal 1–100 range for all 14 skin scores. No record was excluded solely by the positivity requirement. All 387,859 account IDs included in the present

study were represented among the 406,338 unique account IDs in the previous analytic cohort. Thus, at the account-ID level, the present cohort was a nested subset comprising 95.5% of the unique account IDs included in the previous report. The earlier report examined univariable associations between BMI and each of the 14 scores, with detailed analyses centered on redness. The present study instead evaluated the 14-score panel jointly for BMI and BIA-derived outcomes, quantified order-dependent increments after redness was entered first and residual predictive information after BMI inclusion, and assessed performance in a later internal temporal evaluation cohort with no account-ID overlap.

Only measurement month, rather than full date and time, was available in the common source extract. For both the present analysis and the deduplication sensitivity analysis of the previous report, eligible records were ordered by account ID and measurement month, with source-file order used to resolve multiple eligible records occurring within the same month. The first eligible record under this deterministic ordering was retained for each account ID. In the previous analysis, the Pearson correlation between BMI and reverse-coded redness score was materially unchanged after deduplication (all records: n=410,384, r=0.3120; deduplicated: n=406,338, r=0.3131; Δr=0.0011). For the obesity comparisons, the analysis set included 353,925 records before and 350,748 records after deduplication. Repeating the previously reported age-adjusted logistic model with BMI 18.5–24.9 kg/m² as the reference, the odds ratio for high image-derived facial redness (native redness score ≤70; reverse-coded score ≥31) associated with BMI 25.0–29.9 kg/m² changed from 3.146 (95% CI, 3.023–3.275) to 3.141 (95% CI, 3.018–3.270). The corresponding odds ratio for BMI ≥30.0 kg/m² changed from 5.914 (95% CI, 5.604–6.241) to 5.918 (95% CI, 5.607–6.246). The absolute changes were 0.005 and 0.004, respectively. These negligible differences indicate that repeated-record inclusion did not materially affect the previous report's principal association estimates.

Model development and internal temporal evaluation

We fit M0 (age category and recorded sex), MR (M0 plus redness), and M2 (M0 plus all 14 skin scores) using LightGBM regression. [17] Base settings used a learning rate of 0.05, 63 leaves, a minimum of 100 observations per leaf, and deterministic column-wise fitting. Up to 1,000 trees were considered; early stopping after 50 rounds without validation improvement selected the number of trees separately for each body-composition outcome and model. The selected models were refitted using all development account IDs and applied without retuning to the internal temporal evaluation sample. The selected number of trees for each final model is reported in Supplementary Table S1.

Point estimates were calculated in the full internal temporal evaluation cohort (n=159,726). Percentile 95% CIs were obtained from 1,000 paired bootstrap samples. Because each evaluation row represented one deduplicated account ID, row-level resampling was equivalent to account-ID-level resampling. Within each replicate, the same sampled rows were applied to predictions from all compared models, preserving pairing when estimating differences in R^2. These CIs were conditional on the fitted models and did not include model-development uncertainty.

Mean absolute TreeSHAP contributions were computed from each final M2 model using a random sample of 10,000 account IDs from the internal temporal evaluation cohort [18]. Calibration slope and intercept were estimated in the full internal temporal evaluation cohort by unweighted ordinary least squares regression of observed outcomes on predicted values at the individual-record level. Mean calibration error was calculated separately as the unweighted mean of the absolute differences between mean predicted and mean observed values across 10 equal-frequency quantiles of the predicted distribution. Because the 14 skin scores were correlated, TreeSHAP values were used only to describe model reliance and rank features within each fitted model. They do not quantify independent biological contributions, unique information attributable to individual scores, or causal effects, and attribution may be shared or redistributed among correlated features. Calibration slopes are dimensionless; intercepts and mean calibration errors are expressed in the corresponding outcome units. A perfectly calibrated model has a slope of 1.0, an intercept of 0.0, and a mean calibration error of 0. Ties at quantile boundaries could reduce the number of groups, although all three outcomes yielded 10 groups.

In exploratory BMI-informed analyses, baseline LightGBM models for body fat percentage and visceral fat level score included age category, recorded sex, and BMI. Corresponding expanded models additionally included all 14 facial skin scores. Models were developed using April–July data and applied without retuning to the August–October internal temporal evaluation cohort. Differences in R^2 were estimated using 1,000 paired bootstrap samples. Detailed performance results and selected numbers of trees are presented in Supplementary Table S2.

Directional association analysis

To estimate the direction of association independently of TreeSHAP magnitude, 42 separate standardized linear regression models were fitted, with one body-composition outcome as the dependent variable and one facial skin score as the predictor. Both the outcome and skin score were standardized. Models were adjusted for 16

age-category-by-recorded-sex strata and measurement-month fixed effects, and HC1 robust 95% CIs were calculated. P values were adjusted across all 42 tests using the Benjamini–Hochberg false discovery rate procedure. These analyses were considered descriptive and did not establish independent or causal effects because the skin scores were correlated. Complete results are presented in Supplementary Table S3.

Correlations among facial skin scores

Spearman correlations among the 14 facial skin scores were calculated in the internal temporal evaluation cohort (n=159,726). All 14 scores were complete by cohort eligibility. Average ranks were assigned to tied values. The complete correlation matrix is presented in Supplementary Figure S1.

Distributional and sensitivity analyses

Skin-score distributions were summarized using observed ranges, means, standard deviations, medians, interquartile ranges, proportions at the observed maximum, and skewness. To assess robustness to upper-end heaping and nonnormality of redness, a rank-based inverse normal transformation using Blom's formula was applied within the evaluation cohort, with average ranks assigned to ties. Separate linear models evaluated BMI, body fat percentage, and visceral fat level score as dependent variables and transformed redness as the predictor, adjusting for age category and recorded sex. M0 and M2 performance was also estimated separately within recorded-sex strata using paired bootstrap CIs. Sensitivity-analysis results are presented in Supplementary Table S5. The rank-based transformation changed the marginal scale of redness but did not eliminate tied values at the observed maximum. It also did not address heaping or restricted support in the other skin scores. These distributional features may influence tree-split selection and descriptive feature rankings and should not be interpreted as evidence of greater biological importance.

Supplementary Results

Correlations and directional associations

Correlations among the 14 facial skin scores ranged from −0.175 to 0.839 in the internal temporal evaluation cohort. The strongest correlation was observed between texture and moisture (Spearman ρ=0.839;

Supplementary Figure S1). After Benjamini–Hochberg correction across 42 directional tests, 41 associations remained statistically significant. The association between eye-bag score and visceral fat level score was the only exception (FDR-adjusted P=.81; Supplementary Table S3). Redness showed the largest absolute standardized association with BMI (β=−0.258), body fat percentage (β=−0.216), and visceral fat level score (β=−0.172). Given the large sample, statistical significance was not interpreted as evidence of scientific or practical importance. Because lower native redness scores indicate greater image-derived facial redness, these negative coefficients correspond to higher adiposity measures among participants with greater image-derived facial redness.

BMI-informed analysis

In the internal temporal evaluation cohort, the BMI-informed baseline model explained 86.93% of the variance in body fat percentage and 96.16% of the variance in visceral fat level score. Adding all 14 skin scores increased R^2 by 0.00165 (95% CI, 0.00142–0.00187) for body fat percentage and 0.00017 (95% CI, 0.00005–0.00029) for visceral fat level score. MAE decreased from 2.194 to 2.183 percentage points for body fat percentage but increased from 0.526 to 0.532 device-score units (Supplementary Table S2).

Calibration

Calibration slopes for the final M2 models were 1.016 for BMI, 1.008 for body fat percentage, and 1.016 for visceral fat level score. Corresponding calibration intercepts were −0.262, −0.202, and −0.056, respectively. Mean calibration errors across prediction deciles ranged from 0.058 to 0.088 (Supplementary Table S4 and Supplementary Figure S2).

In the evaluation sample (n=159,726), higher RINT-transformed native redness score was associated with lower BMI (β, −1.022; 95% CI, −1.042 to −1.002), body fat percentage (β, −1.817; 95% CI, −1.854 to −1.778), and visceral fat level score (β, −0.914; 95% CI, −0.931 to −0.899), consistent with the direction of the native-score analyses.

In recorded-sex-stratified analyses, M2−M0 ΔR^2 values among female participants were 0.1461 (95% CI, 0.1411–0.1515) for BMI, 0.1229 (0.1189–0.1269) for body fat percentage, and 0.1191 (0.1153–0.1234) for

visceral fat level score. Corresponding estimates among male participants were 0.1375 (0.1320–0.1427), 0.1022 (0.0979–0.1066), and 0.0892 (0.0856–0.0928), respectively.

Supplementary Table S1. Selected numbers of trees for the final LightGBM models

Outcome	Model	Predictor set	Selected trees
BMI (kg/m²)	M0	Age category + recorded sex	480
BMI (kg/m²)	MR	Age category + recorded sex + redness	86
BMI (kg/m²)	M2	Age category + recorded sex + all 14 skin scores	326
Body fat percentage (%)	M0	Age category + recorded sex	94
Body fat percentage (%)	MR	Age category + recorded sex + redness	85
Body fat percentage (%)	M2	Age category + recorded sex + all 14 skin scores	297
Body fat percentage (%)	BMI-informed baseline	Age category + recorded sex + BMI	111
Body fat percentage (%)	BMI-informed expanded	Age category + recorded sex + BMI + 14 skin scores	233
Visceral fat level score	M0	Age category + recorded sex	448
Visceral fat level score	MR	Age category + recorded sex + redness	99
Visceral fat level score	M2	Age category + recorded sex + all 14 skin scores	224
Visceral fat level score	BMI-informed baseline	Age category + recorded sex + BMI	171
Visceral fat level score	BMI-informed expanded	Age category + recorded sex + BMI + 14 skin scores	250

Note: M0 included age category and recorded sex; MR additionally included the native redness score; M2 included age category, recorded sex, and all 14 facial skin scores. BMI-informed baseline models included age category, recorded sex, and BMI; BMI-informed expanded models additionally included all 14 facial skin scores. The number of trees was selected separately for each outcome and model by early stopping on the development validation set.

Supplementary Table S2. Performance of exploratory BMI-informed models in the internal temporal evaluation cohort

Outcome	Model	n	R^2	MAE	ΔR^2 vs baseline (95% CI)	Selected trees
Body fat percentage (%)	Baseline	159,726	0.86927	2.194	—	111
Body fat percentage (%)	Expanded	159,726	0.87092	2.183	0.00165 (0.00142 to 0.00187)	233
Visceral fat level score	Baseline	159,726	0.96156	0.526	—	171
Visceral fat level score	Expanded	159,726	0.96173	0.532	0.00017 (0.00005 to 0.00029)	250

Note: Baseline models included age category, recorded sex, and BMI; expanded models additionally included all 14 facial skin scores. Models developed using April–July 2025 data were applied without retuning to the August–October 2025 internal temporal evaluation cohort. Percentile 95% confidence intervals were obtained from 1,000 paired account-ID bootstrap samples. MAE is expressed in percentage points for body fat percentage and device-score units for visceral fat level score. Abbreviations: CI, confidence interval; MAE, mean absolute error.

Supplementary Table S3. Adjusted directional associations between individual facial skin scores and body-composition outcomes

Outcome	Facial skin score	n	Standardized β (95% CI)	Raw P value	FDR-adjusted P value
BMI (kg/m²)	Redness	159,726	−0.258 (−0.262 to −0.253)	<0.001	<0.001
BMI (kg/m²)	Texture	159,726	−0.158 (−0.164 to −0.152)	<0.001	<0.001
BMI (kg/m²)	Moisture	159,726	−0.155 (−0.161 to −0.149)	<0.001	<0.001
BMI (kg/m²)	Radiance	159,726	−0.132 (−0.139 to −0.126)	<0.001	<0.001
BMI (kg/m²)	Upper eyelid sagging	159,726	−0.098 (−0.104 to −0.093)	<0.001	<0.001
BMI (kg/m²)	Pigmentation	159,726	−0.098 (−0.104 to −0.092)	<0.001	<0.001
BMI (kg/m²)	Acne	159,726	−0.078 (−0.083 to −0.073)	<0.001	<0.001
BMI (kg/m²)	Dark circles	159,726	0.075 (0.070 to 0.080)	<0.001	<0.001
BMI (kg/m²)	Oiliness	159,726	0.068 (0.063 to 0.073)	<0.001	<0.001
BMI (kg/m²)	Firmness	159,726	0.051 (0.044 to 0.057)	<0.001	<0.001
BMI (kg/m²)	Wrinkles	159,726	−0.051 (−0.058 to −0.043)	<0.001	<0.001
BMI (kg/m²)	Lower eyelid sagging	159,726	0.043 (0.038 to 0.047)	<0.001	<0.001
BMI (kg/m²)	Pores	159,726	−0.027 (−0.033 to −0.022)	<0.001	<0.001
BMI (kg/m²)	Eye bag	159,726	0.023 (0.017 to 0.030)	<0.001	<0.001
Body fat percentage (%)	Redness	159,726	−0.216 (−0.220 to −0.211)	<0.001	<0.001
Body fat percentage (%)	Texture	159,726	−0.114 (−0.120 to −0.109)	<0.001	<0.001
Body fat percentage (%)	Moisture	159,726	−0.108 (−0.114 to −0.103)	<0.001	<0.001
Body fat percentage (%)	Radiance	159,726	−0.086 (−0.092 to −0.080)	<0.001	<0.001
Body fat percentage (%)	Upper eyelid sagging	159,726	−0.080 (−0.085 to −0.075)	<0.001	<0.001
Body fat percentage (%)	Pigmentation	159,726	−0.075 (−0.080 to −0.069)	<0.001	<0.001
Body fat percentage (%)	Acne	159,726	−0.071 (−0.075 to −0.066)	<0.001	<0.001
Body fat percentage (%)	Firmness	159,726	0.065 (0.059 to 0.072)	<0.001	<0.001
Body fat percentage (%)	Oiliness	159,726	0.063 (0.059 to 0.067)	<0.001	<0.001
Body fat percentage (%)	Dark circles	159,726	0.062 (0.057 to 0.066)	<0.001	<0.001
Body fat percentage (%)	Lower eyelid sagging	159,726	0.021 (0.016 to 0.025)	<0.001	<0.001

Body fat percentage (%)	Eye bag	159,726	0.019 (0.013 to 0.025)	<0.001	<0.001
Body fat percentage (%)	Pores	159,726	−0.015 (−0.020 to −0.010)	<0.001	<0.001
Body fat percentage (%)	Wrinkles	159,726	−0.012 (−0.019 to −0.006)	<0.001	<0.001
Visceral fat level score	Redness	159,726	−0.172 (−0.175 to −0.169)	<0.001	<0.001
Visceral fat level score	Moisture	159,726	−0.115 (−0.119 to −0.111)	<0.001	<0.001
Visceral fat level score	Texture	159,726	−0.110 (−0.115 to −0.106)	<0.001	<0.001
Visceral fat level score	Radiance	159,726	−0.083 (−0.087 to −0.078)	<0.001	<0.001
Visceral fat level score	Upper eyelid sagging	159,726	−0.069 (−0.072 to −0.065)	<0.001	<0.001
Visceral fat level score	Pigmentation	159,726	−0.064 (−0.068 to −0.060)	<0.001	<0.001
Visceral fat level score	Oiliness	159,726	0.054 (0.050 to 0.057)	<0.001	<0.001
Visceral fat level score	Acne	159,726	−0.050 (−0.054 to −0.047)	<0.001	<0.001
Visceral fat level score	Dark circles	159,726	0.043 (0.040 to 0.047)	<0.001	<0.001
Visceral fat level score	Wrinkles	159,726	−0.040 (−0.045 to −0.035)	<0.001	<0.001
Visceral fat level score	Firmness	159,726	0.037 (0.032 to 0.042)	<0.001	<0.001
Visceral fat level score	Lower eyelid sagging	159,726	0.023 (0.019 to 0.026)	<0.001	<0.001
Visceral fat level score	Pores	159,726	−0.018 (−0.021 to −0.014)	<0.001	<0.001
Visceral fat level score	Eye bag	159,726	−0.001 (−0.005 to 0.004)	0.813	0.813

Note: Each row represents a separate linear model with the body-composition outcome as the dependent variable and one facial skin score as the predictor. The outcome and skin score were standardized. Models were adjusted for 16 age-category-by-recorded-sex strata and measurement-month fixed effects, with HC1 robust 95% confidence intervals. P values were adjusted across all 42 tests using the Benjamini–Hochberg false discovery rate procedure. Higher skin scores indicate a more favorable skin condition; a lower redness score indicates greater image-derived facial redness. Abbreviations: CI, confidence interval; FDR, false discovery rate.

Supplementary Table S4. Calibration of the final M2 models in the internal temporal evaluation cohort

Outcome	n	Calibration slope	Calibration intercept	Mean calibration error
BMI (kg/m²)	159,726	1.016	−0.262	0.088
Body fat percentage (%)	159,726	1.008	−0.202	0.058
Visceral fat level score	159,726	1.016	−0.056	0.061

Note: Calibration slope and intercept were estimated by unweighted ordinary least squares regression of observed outcomes on model predictions at the individual-record level in the full internal temporal evaluation cohort. Mean calibration error was calculated separately as the unweighted mean of the absolute differences between mean predicted and mean observed values across 10 equal-frequency quantiles of predicted values. Calibration slopes are dimensionless. Intercepts and mean calibration errors are expressed in kg/m² for BMI, percentage points for body fat percentage, and device-score units for the visceral fat level score.

Supplementary Table S5. Sensitivity analyses

Panel A. Model performance among account IDs with one eligible record

Outcome	Model	n	R^2	MAE	M2−M0 ΔR^2
BMI (kg/m²)	M0	159,125	0.12514	2.712	—
BMI (kg/m²)	M2	159,125	0.25601	2.500	0.13087
Body fat percentage (%)	M0	159,125	0.25447	5.373	—
Body fat percentage (%)	M2	159,125	0.34703	5.034	0.09256
Visceral fat level score	M0	159,125	0.54910	2.198	—
Visceral fat level score	M2	159,125	0.61236	2.043	0.06325

Note: This analysis excluded 601 evaluation-cohort account IDs that had more than one eligible record and retained 159,125 singleton account IDs. The previously fitted frozen models were applied without refitting. Point estimates are reported; confidence intervals were not calculated for this sensitivity analysis.

Panel B. Rank-based inverse normal transformation sensitivity analysis for redness

Outcome	n	Regression coefficient (95% CI)	R^2
BMI (kg/m²)	159,726	−1.022 (−1.042 to −1.002)	0.18347
Body fat percentage (%)	159,726	−1.817 (−1.854 to −1.778)	0.29592
Visceral fat level score	159,726	−0.914 (−0.931 to −0.899)	0.55271

Note: Native redness scores were transformed using Blom's rank-based inverse normal transformation with average ranks for ties. Coefficients represent the change in the outcome's native units per 1-unit increase in transformed redness, adjusted for age category and recorded sex. A negative coefficient indicates a lower body-composition outcome at a higher, more favorable native redness score. Abbreviation: CI, confidence interval.

Panel C. Recorded-sex-stratified model performance

Outcome	Recorded sex	n	M0 R^2	M2 R^2	M2−M0 ΔR^2 (95% CI)
BMI (kg/m²)	Male	60,875	0.05833	0.19581	0.13748 (0.13197 to 0.14271)
BMI (kg/m²)	Female	98,851	0.03399	0.18004	0.14605 (0.14106 to 0.15150)
Body fat percentage (%)	Male	60,875	0.10741	0.20963	0.10222 (0.09789 to 0.10660)
Body fat percentage (%)	Female	98,851	0.03270	0.15562	0.12292 (0.11890 to 0.12694)
Visceral fat level score	Male	60,875	0.34928	0.43847	0.08919 (0.08560 to 0.09277)
Visceral fat level score	Female	98,851	0.17487	0.29400	0.11913 (0.11530 to 0.12338)

Note: Models were estimated separately within each recorded-sex stratum using the primary M0 and M2 predictor definitions. Percentile 95% confidence intervals for M2−M0 ΔR^2 were obtained from paired bootstrap samples. Subgroup differences were descriptive and were not formally tested as interactions. Abbreviation: CI, confidence interval.

Supplementary Figure S1. Spearman correlation matrix of the 14 facial skin scores in the internal temporal evaluation cohort. For the native redness score, lower values indicate greater image-derived facial redness.

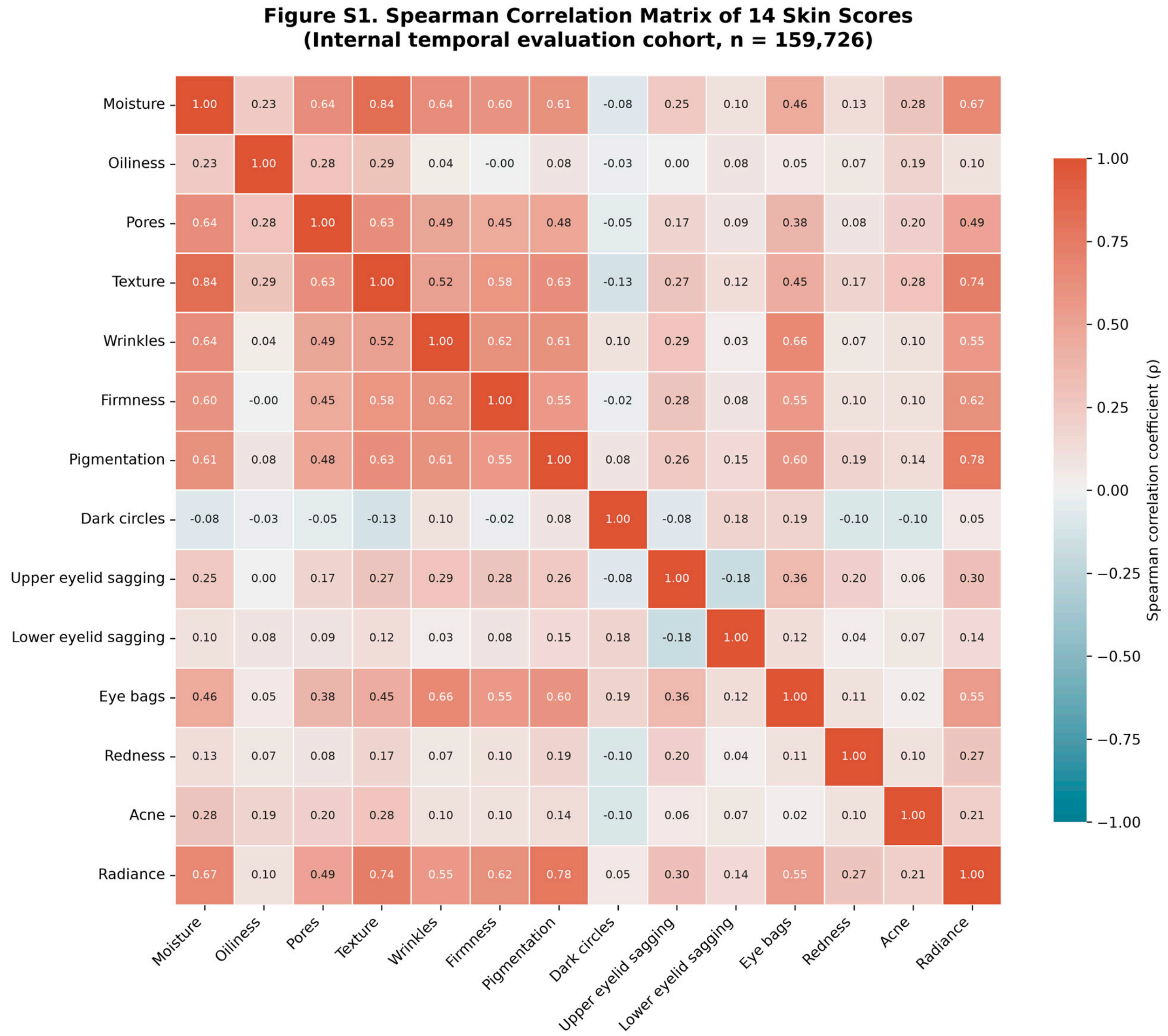

Values represent Spearman correlation coefficients calculated in 159,726 account IDs. Average ranks were assigned to tied values.

Supplementary Figure S2. Calibration of the final M2 models in the internal temporal evaluation cohort.

Figure S2. Calibration Plots for M2 Model Predictions
(Internal temporal evaluation cohort, n = 159,726)

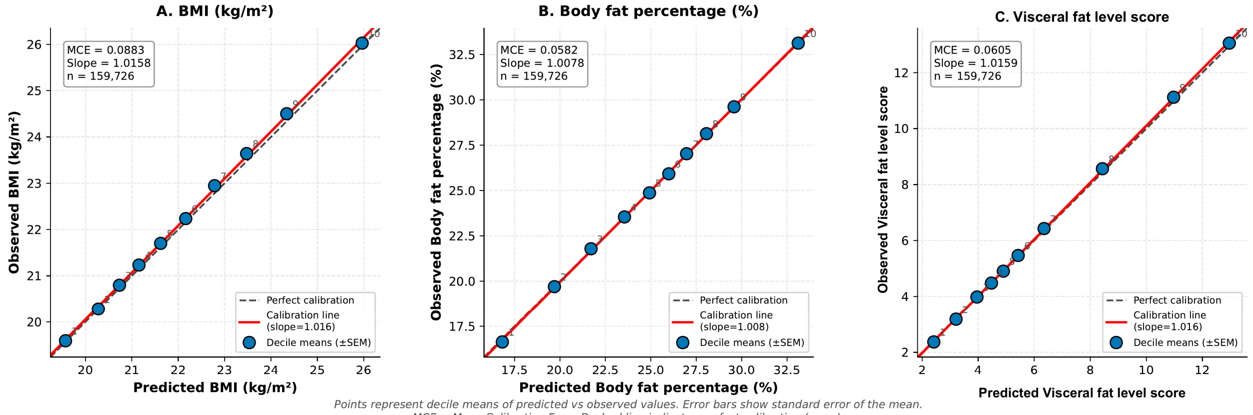

Points represent decile means of predicted vs observed values. Error bars show standard error of the mean.
MCE = Mean Calibration Error. Dashed line indicates perfect calibration (y = x).

Mean observed outcomes are plotted against mean predictions within deciles of predicted values for (A) BMI, (B) body fat percentage, and (C) visceral fat level score. The dashed diagonal line represents perfect agreement. The evaluation cohort included 159,726 account IDs. The visceral fat level score is a proprietary BIA-derived index and not a direct measurement of visceral adipose tissue.

Ethics

The study was conducted in accordance with the Declaration of Helsinki and approved by the Ethics Committee of Kindai University Faculty of Medicine (approval no. R07-210; February 2, 2026). The supplied research extract excluded individuals younger than 18 years. All included participants had provided informed consent at the time of data collection, and the investigators received a fully anonymized dataset containing no identifiable personal information.

Supplementary References

Reference numbers correspond to those in the main manuscript.

10. Katragadda, C. *et al.* Technique standards for skin lesion imaging: A Delphi consensus statement. *JAMA Dermatol.* **153**, 207–213 (2017).

11. Daneshjou, R. *et al.* Checklist for evaluation of image-based artificial intelligence reports in dermatology: CLEAR derm consensus guidelines from the International Skin Imaging Collaboration artificial intelligence working group. *JAMA Dermatol.* **158**, 90–96 (2022).

12. Iinuma, K. et al. AI-derived facial redness score is associated with body mass index in a large Japanese screening cohort. JAAD Int. 28, 116-117 (2026).

14. Kyle, U. G. *et al.* Bioelectrical impedance analysis--part I: review of principles and methods. *Clin. Nutr.* **23**, 1226–1243 (2004).

15. Murphy, J., Bacon, S. L., Morais, J. A., Tsoukas, M. A. & Santosa, S. Intra-abdominal adipose tissue quantification by alternative versus reference methods: A systematic review and meta-analysis. Obesity (Silver Spring) 27, 1115–1122 (2019).

17. Ke G, Meng Q, Finley T, et al. LightGBM: a highly efficient gradient boosting decision tree. Adv Neural Inf Process Syst.30:3146-3154. (2017).

18. Lundberg, S. M. *et al.* From local explanations to global understanding with explainable AI for trees. *Nat. Mach. Intell.* **2**, 56–67 (2020).
